# Acrylonitrile Butadiene Styrene and Polypropylene Blend with Enhanced Thermal and Mechanical Properties for Fused Filament Fabrication

**DOI:** 10.3390/ma12244167

**Published:** 2019-12-11

**Authors:** Muhammad Harris, Johan Potgieter, Sudip Ray, Richard Archer, Khalid Mahmood Arif

**Affiliations:** 1 Department of Mechanical and Electrical Engineering, SF&AT, Massey University, Auckland 0632, New Zealand; m.harris@massey.ac.nz; 2 School of Food and Advanced Technology, Massey University, Palmerston North 4442, New Zealand; j.potgieter@massey.ac.nz (J.P.); r.h.archer@massey.ac.nz (R.A.); 3 Department of Chemical Sciences, The University of Auckland, Auckland 1010, New Zealand; s.ray@auckland.ac.nz

**Keywords:** fused filament fabrication, three-dimensional (3D) printing materials, acrylonitrile butadiene styrene, polypropylene, polyethylene graft maleic anhydride, thermal degradation

## Abstract

Acrylonitrile butadiene styrene (ABS) is the oldest fused filament fabrication (FFF) material that shows low stability to thermal aging due to hydrogen abstraction of the butadiene monomer. A novel blend of ABS, polypropylene (PP), and polyethylene graft maleic anhydride (PE-g-MAH) is presented for FFF. ANOVA was used to analyze the effects of three variables (bed temperature, printing temperature, and aging interval) on tensile properties of the specimens made on a custom-built pellet printer. The compression and flexure properties were also investigated for the highest thermal combinations. The blend showed high thermal stability with enhanced strength despite three days of aging, as well as high bed and printing temperatures. Fourier-transform infrared spectroscopy (FTIR) provided significant chemical interactions. Differential scanning calorimetry (DSC) confirmed the thermal stability with enhanced enthalpy of glass transition and melting. Thermogravimetric analysis (TGA) also revealed high temperatures for onset and 50% mass degradation. Signs of chemical grafting and physical interlocking in scanning electron microscopy (SEM) also explained the thermo-mechanical stability of the blend.

## 1. Introduction

Acrylonitrile butadiene styrene (ABS) is one of the common terpolymers in conventional polymer processes and fused filament fabrication (FFF) [1,2]. It is composed of styrene acrylonitrile (SAN) grafted with polybutadiene (PB) that provides diverse properties (tensile strength, ductility, hydrophobicity) to suit a wide range of applications in conventional polymer processes [3]. Meanwhile, it is one of the oldest FFF materials that is used to three-dimensionally (3D) print a wide range of additive manufacturing (AM) applications, such as prototypes, airfoils, clamps, etc. The recent application of ABS with carbon fibers in big area additive manufacturing (BAAM) re-defined its importance for large-scale manufacturing [4,5]. The large-scale applications demand enhanced stability that can withstand high applied loads at high temperatures. Although ABS has good mechanical, chemical, and thermal properties at room temperature [6], the hydrogen abstraction from PB at high temperatures results in a decline in thermal stability [7,8]. The hydrogen abstraction reported a significant impact on the mechanical and thermal properties due to the different weathering conditions above 40 °C [7,9,10]. Therefore, a need arises to explore a solution for a thermally stable ABS for large-scale and high temperature FFF applications.

Different methods were adopted to increase the thermal stability of ABS [11,12], such as blending [11,12], addition of flame retardants [13], stabilizers [11], etc. The most recommended method in terms of gaining both thermal and mechanical stability is blending with high-temperature polymers [12,13,14]. In this regard, polycarbonate (PC) is the most successful polymer that reports the best-in-class blend properties with ABS [14] as compared to contemporary ones in FFF, such as ABS/UHMWPE/SEBS, ABS:SEBS [15,16], ABS:SEBS-g-MAH [17], ABS/PMMA [18], and ABS/SMA [5]. The addition of PC to ABS was reported with improvements in both thermal and mechanical properties (tensile, compression, and flexure) [13,19]. However, thermal improvement is interpreted as an improvement in the onset of the degradation temperature of the non-aged blend obtained in thermogravimetric analysis (TGA) [20]. Researchers found detrimental effects of annealing on the morphology of an ABS/PC blend that resulted in a decrease in thermal stability [20]. Therefore, it reveals a need to explore a blend of ABS that can withstand thermal aging at high temperatures with good mechanical properties (tensile, compression, and flexure).

Polypropylene (PP) is one of the eminent polyolefins with good chemical and mechanical properties [21]. However, it presents warpage and swelling in an FFF process. This is overcome by blending with different printable polymers or fillers (fibers). For example, Peng et al. [22] reported enhanced mechanical strength with the addition of polyamide (PA6) and POE-g-MAH. Ramis et al. [21] reported enhanced thermal stability in the TGA analysis of PP/starch. Mourad et al. [23] found good resistance to thermal aging and unaffected tensile strength in a PP/PE blend. Carneiro et al. [24] and Sodeifian et al. [25] reported improved mechanical strength with the addition of glass fibers in PP. It is noted that the proposed blends with or without fillers provided either good mechanical strength or resistance to aging. Furthermore, the mechanical characterization of the blends is mostly limited to either tensile or flexural strength. The improved overall mechanical stability (tensile, compressive, and flexural) at higher temperatures (thermal aging) for numerous days is still not reported in the FFF literature for PP blends.

This research proposes a novel FFF blend of ABS with PP that can withstand thermal aging with good mechanical properties (tensile, compressive and flexure). Polyethylene graft maleic anhydride (PE-g-MAH) is used to compatibilize both polymers. A statistical design of experiments (DoE) constituting33 full factorials is designed to analyze the tensile properties and thermal stability against three variables (bed temperature, printing temperature, and aging interval). The compression and flexural experimentations are performed at three such combinations from the ANOVA analysis with the highest levels of thermal variables. The blend is further investigated by Fourier-transform infrared spectroscopy (FTIR), differential scanning calorimetry (DSC), thermogravimetric analysis (TGA), and scanning electron microscopy (SEM) to ascertain the reasons for the improved thermo-mechanical properties. 

## 2. Materials and Methods

### 2.1. Materials

Polylac PA-747 ABS pellets with a high melt flow index of 13 g/10 min were purchased from TCL Hunt (Auckland, New Zealand). LeyondellBasell PP with a melt flow index of 10 g/10 min was also purchased from TCL Hunt. A8525 PE-g-MAH (compatibilizer) was purchased from Shenzhen Jindaquan Technology Co. Ltd. (Shenzhen, China).

### 2.2. Blending

ABS, PP, and PE-g-MAH were dried at 50 °C in an oven for six hours. The drying was followed by mixing all three polymers in specific compositions in a 5-L Haake mixer (Thermo Fisher, Waltham, MA, USA) for five minutes. The compositions were blended in a single-screw extruder (Beierman, Leyuzhen, China), which was specifically selected for blending in order to avoid any probable degradation due to shearing pressure at high temperatures [26]. The pellets were produced in a size of approximately 1.3 mm with the Beierman pelletizer unit operating at 20 m/min. Further processing parameters are provided in Table 1.

Successful 3D printing was one of the main objectives in this research. The compositions of each of the three polymers in the blend can potentially affect the printability. For example, Zhang et al. [27] reported defects in rheological properties due to an excess of maleic anhydride (MAH). Rheological defects can lead to serious problems in the printing. On the other hand, the high composition of PP was reported to cause large die swelling during extrusion [28] and warpage during printing [24,29]. Therefore, the first blend was prepared with 48:48 by weight percentage of ABS/PP (Table 2) with 4% PE-g-MAH [30,31], whose printing failed. For the second and third compositions (Table 2), the composition of PP was decreased while keeping the compatibilizer the same at 4%. However, failed printing for the second and partial printing with high warpage for the third composition led to the preparation of a fourth composition. The fourth composition, prepared with 0.5% PE-g-MAH [32], provided the best printing. Therefore, no further composition needed to be prepared.

### 2.3. 3D printing

**Figure 1:**
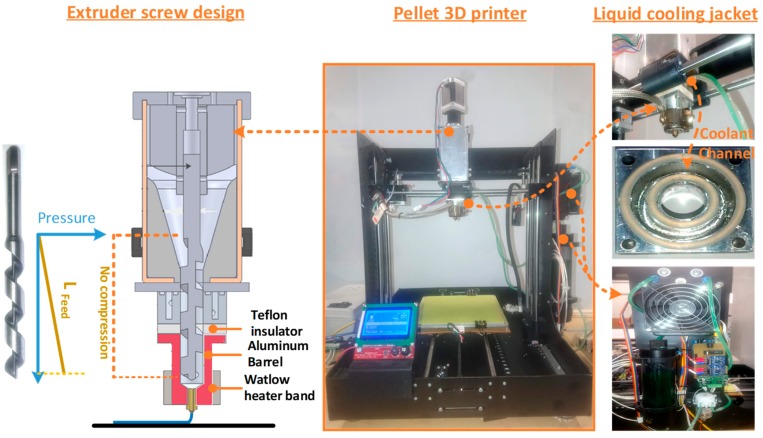
Illustration of different parts of pellet three-dimensional (3D) printer [33] and the modifications in extruder screw and cooling system.

The 3D printing was performed on our in-house-built pellet 3D printer (Figure 1) that was previously reported [33]. Modifications were particularly made in the cooling system, insulation, and extruder screw to avoid variations in the material properties during printing. The liquid cooling system was improved with an enlarged liquid channel in the liquid cooling jacket. A “Teflon insulation” plate was included as a thermal barrier. Both the liquid cooling system and the Teflon plate improved the resistance to the heat transfer through the extruder. The better resistance helped to enable printing at high temperatures above 200 °C. Furthermore, the extruder screw design was refined (Figure 1) with the exclusion of the compression and metering zones unlike regular screw extruders. The screw extruder’s feeding zone avoids any thermal compression of the melt. This helps to overcome variations or degradation in the material properties due to any thermal shearing [33,34]. The three main modifications (cooling system, Teflon insulation, and screw extruder) are expected to achieve printing with characteristics as near as possible to the original blend. 

An open source software named “Slic3r” was used to control the printing parameters (Table 3), and “Pronterface” was used to operate the printer.

**Figure 2:**
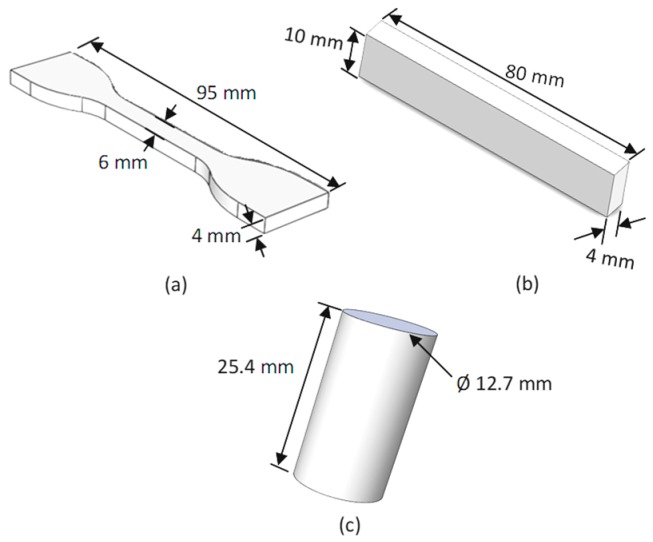
Standards for sample testing: (**a**) ASTM D638 for tensile testing, (**b**) ISO 604 for compression testing, and (**c**) ISO 178 for flexure testing.

**Figure 3:**
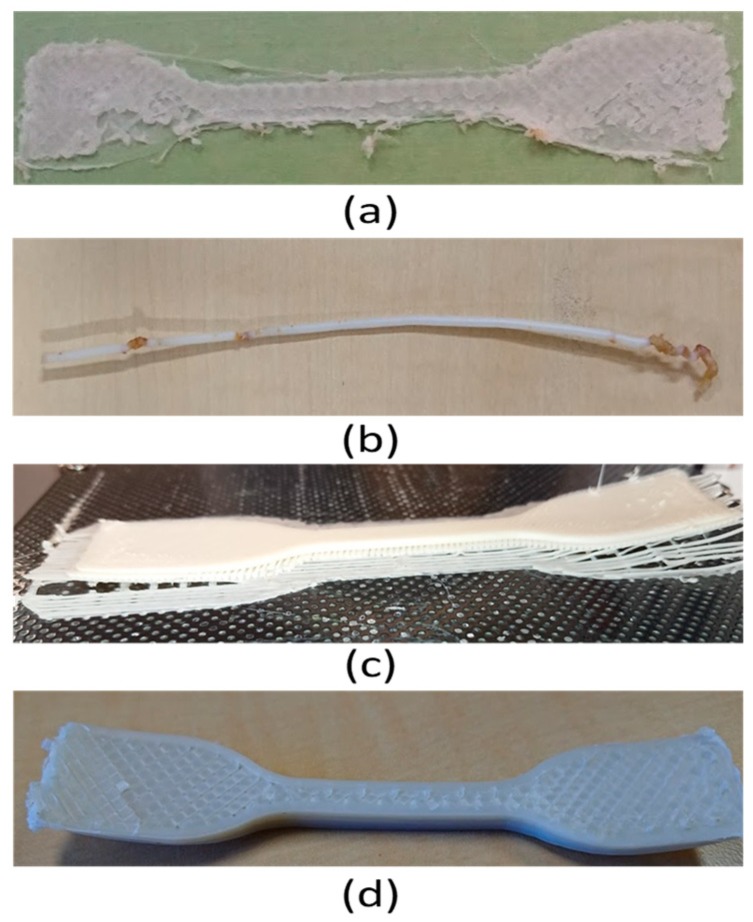
Failed printing (**a**) when printing at 175 °C, (**b**) due to thermal degradation in extruded filament above 210 °C, (**c**) when printing third composition with perforated board, and (**d**) due to deflection after printing using third composition.

ASTM D638 type IV [35] was used as the tensile testing standard, ISO 604 [35] was used for compression, and ISO 178 [36] was used for the flexural testing standard (Figure 2). The first two compositions showed large die swelling during extrusion printing. Furthermore, the extrusion rate during printing was too slow to even meet the lowest printing speed of 1 mm/min. The third composition was partially printed. The excessive warpage caused incomplete printing (Figure 3c,d). Different types of surfaces (perforated, flex) were also used to improve the adhesion of the third composition specimens with the printing bed. However, the sample resulted in high delamination and incomplete printing due to the excessive warpage (Figure 3c). The fourth composition was printed on an adhesion tape printing bed. It showed minimum to no warpage. Therefore, the remaining experimentation of this research was conducted with the fourth composition.

A randomized general 3^3^ full factorial ANOVA analysis in Minitab2019 was performed for tensile testing at a confidence level of 95%. Three variables were selected to analyze the thermal stability on tensile strength. Each variable had three levels, i.e., bed temperature was set at 25 °C, 50 °C, and 75 °C, printing temperature was set at 185 °C, 195 °C, and 205 °C, and aging interval was set at zero days, three days, and six days. An incomplete printing below 178 °C was noted, as the temperature was too low to melt the material. Similarly, printing above 210 °C resulted in burnt marks in the extrudate. Furthermore, the minimum temperature of the bed was set at 25 °C with reference to the room temperature, and the highest temperature of 75 °C in this research was based upon the literature [37,38]. The aging intervals were also selected according to the literature [10]. Three samples were printed for each of the 27 combinations and an average of tensile strength and strain was used in the analysis. However, the analysis was performed with respect to tensile strength, not tensile strain.

Based on the objective of evaluation of thermal stability of the novel FFF material and significance of the bed and printing temperature, the combinations with the highest bed and printing temperatures were used to print and analyze the compressive and flexural properties.

### 2.4. Mechanical Testing

Instron 5967 with a 30-kN load cell was used to perform the mechanical testing (tensile, compression, and flexural). The tensile testing on ASTM D638 type IV dog bones was performed at an extension rate of 5 mm/min with a 25-mm clip-on gauge extensometer for measurement of tensile extension. The flexure testing of ISO 178 rectangular bars was performed on a three-point flexure set-up at a rate of 2 mm/min. The horizontal span between the two loading anvils was set at 64 mm as per ISO 178. The compressive testing of ISO 604 cylindrical samples was performed at 2 mm/min. The variations in the flexural samples of combination 27 were particularly monitored, and the samples with too high dimensional instability were discarded. Similarly, the vertical alignment of layers during printing led to a proper compression. The vertically mis-aligned samples displayed buckling and, therefore, were discarded as well.

### 2.5. Fourier-Transform Infrared Spectroscopy (FTIR)

FTIR was used for analysis of the effects of blending, 3D printing, and thermal aging on a Thermo Electron Nicolet 8700 spectrometer (ThermoFisher, Waltham, MA, USA) with a spectrum range of 400–4000 cm^−1^. OMNIC E.S.P 7.1 was used to collect the data from an attenuated total reflection accessory that provided an average spectrum of a total of 32 scans. All FTIR spectra were corrected and normalized with respect to a baseline.

### 2.6. Differential Scanning Calorimetry (DSC)

The effects of three variables (bed temperature, printing temperature, and aging interval) on the glass crystallization, melt crystallization and degradation were measured with DSC Q1000 (New TA instrument, New Castle, DE, USA) from TA Instruments. The instrument was operated at a heating rate of 10 °C/min in nitrogen gas purged at a flow rate of 50 mL/min. The range of temperature for DSC analysis was 25 °C to 550 °C. 

### 2.7. Thermogravimetric Analysis (TGA)

The effects of experimental variables on the onset temperature and 50% degradation were analyzed on an STA 449 F1 Jupiter (NETZSCH, Selb, Germany). The thermal analysis was conducted at a rate of 10 °C/min in nitrogen gas purged at 50 mL/min. The analysis was performed in a range of 25 °C to 550 °C.

### 2.8. Scanning Electron Microscopy (SEM)

The visual phase separations between ABS and PP at the fractured surfaces were analyzed by an SEM TM3030 Plus (Hitachi, Tokyo, Japan). It was also used to analyze the effects of aging interval, bed temperature, and printing temperature. The images were used to describe the tensile properties in ANOVA analysis.

## 3. Results

### 3.1. Tensile Testing

**Figure 4:**
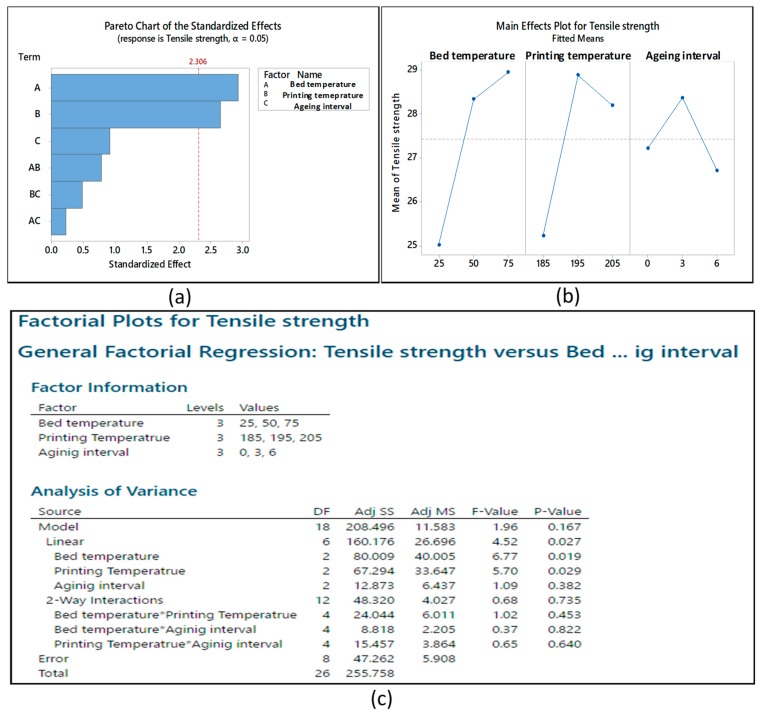
ANOVA analysis: (**a**) Pareto chart, (**b**) main effects plot, and (**c**) *p*-value for main effects and two-way interactions.

The 3^3^ full factorial design of experiment with tensile strength and strain is given in Table 4. ANOVA analysis of the data in Figure 4 shows the bed temperature and printing temperature as the significant variables in a Pareto chart. The interval plots further elaborate the individual effects of each variable on the tensile strength. It is noted that the printing temperature resulted in a decline in strength with a rise from 195 °C to 205 °C. This also explains the burnt marks above 210 °C. However, the decline was not significant. Therefore, it cannot be considered as a degradation of thermal stability at 205 °C.

**Figure 5:**
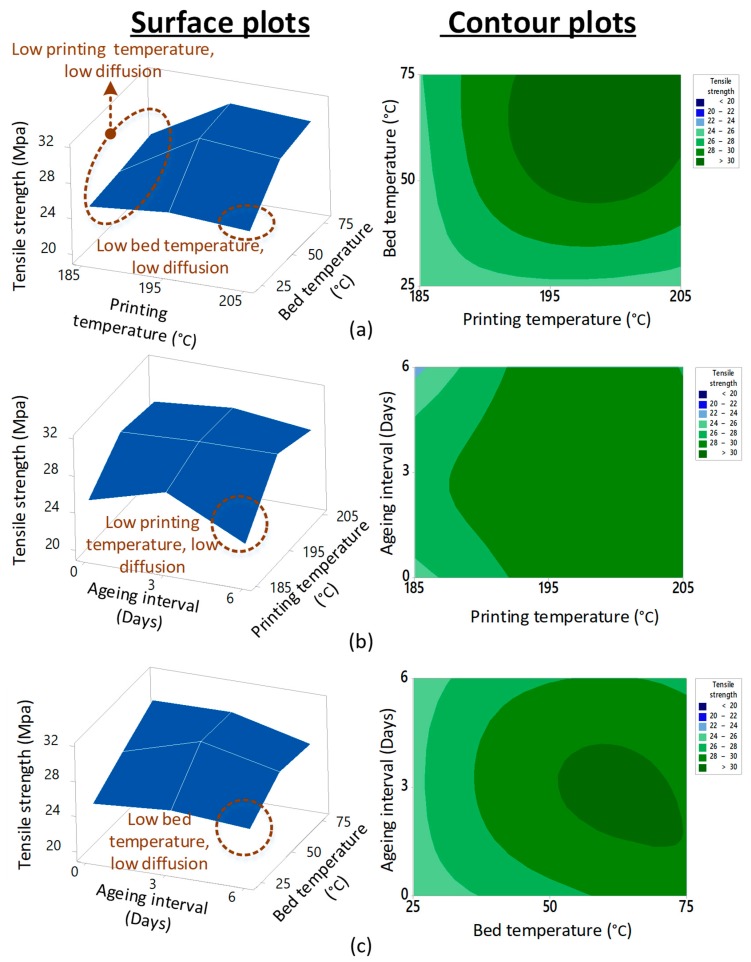
Surface plots for (**a**) printing vs. bed temperature, (**b**) aging interval vs. printing temperature, and (**c**) aging interval vs. bed temperature.

The surface plots and contour plots show the significance of high printing and bed temperature (Figure 5a). The three binary plots (surface and contour) show a decline in tensile strength at the lowest bed temperature (25 °C) and printing temperature (185 °C). The decrease was the result of low diffusion between the beads due to either low bed or printing temperature [39]. It is also noted that, at the highest aging interval (six days), the strength decreased for the lowest printing and bed temperatures and increased for the highest corresponding variables (Figure 5b,c). The increase in tensile properties after six days of aging depicted the non-significance of the aging interval and, hence, the thermal stability was statistically proven based on the non-replicative DoE in this research.

**Figure 6:**
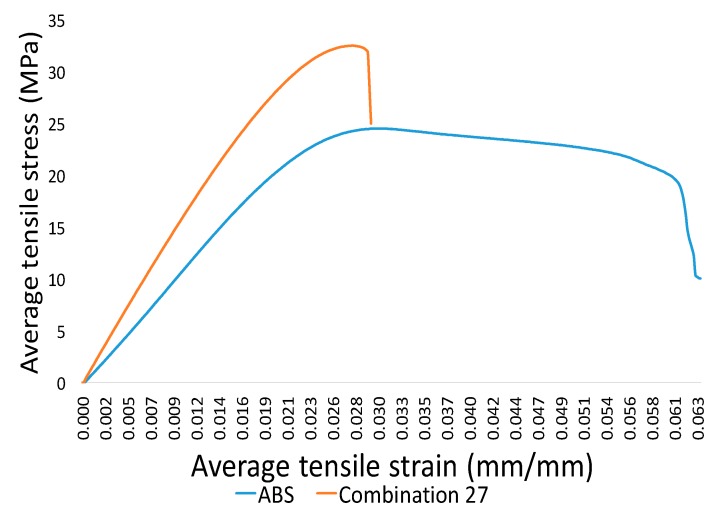
Stress–strain graphs for acrylonitrile butadiene styrene (ABS) and blend (combination 27).

The analysis of stress–strain graphs in Figure 6 shows far higher stiffness for the blend as compared to neat ABS at combination 27 (bed at 75 °C, printing at 205 °C, aging at six days). This shows the improvement in one of the important mechanical characteristics (stiffness) of the novel FFF blend.

### 3.2. Compressive and Flexural Testing

**Figure 7:**
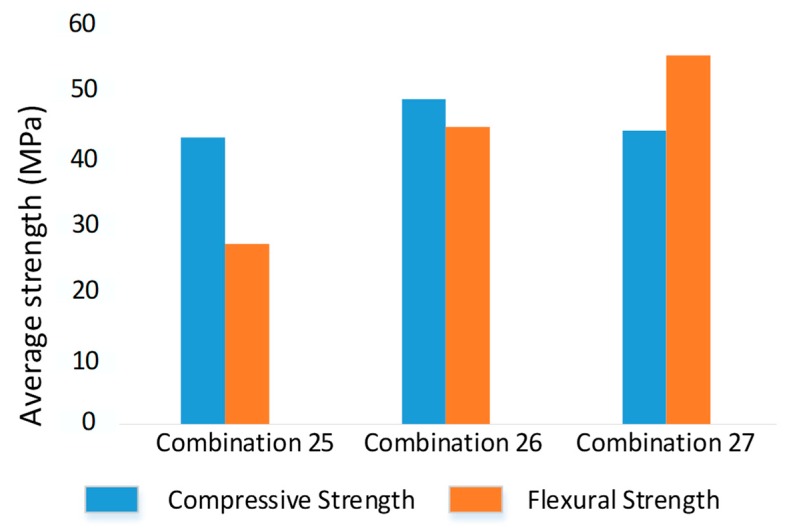
Compression and flexure strength of three combinations at highest bed and printing temperatures.

A similar resistance to thermal aging as for tensile strength was observed for compression and flexure properties (Figure 7). The flexure properties increased to as high as 54.9 MPa with an increase in aging interval. This may be due to the improvement in diffusion between printed layers during aging instead of degradation. The compression strength showed significant enhancement from zero to three days of aging, when the compression properties increased to 48.4 MPa. However, it dropped down to 43.7 MPa after six days of aging. This decrease in compression value after six days seems insignificant considering the number of days, as the drop was just 4.7 MPa.

## 4. Discussion

### 4.1. Effects of Blending, Printing, and Thermal Aging 

**Figure 8:**
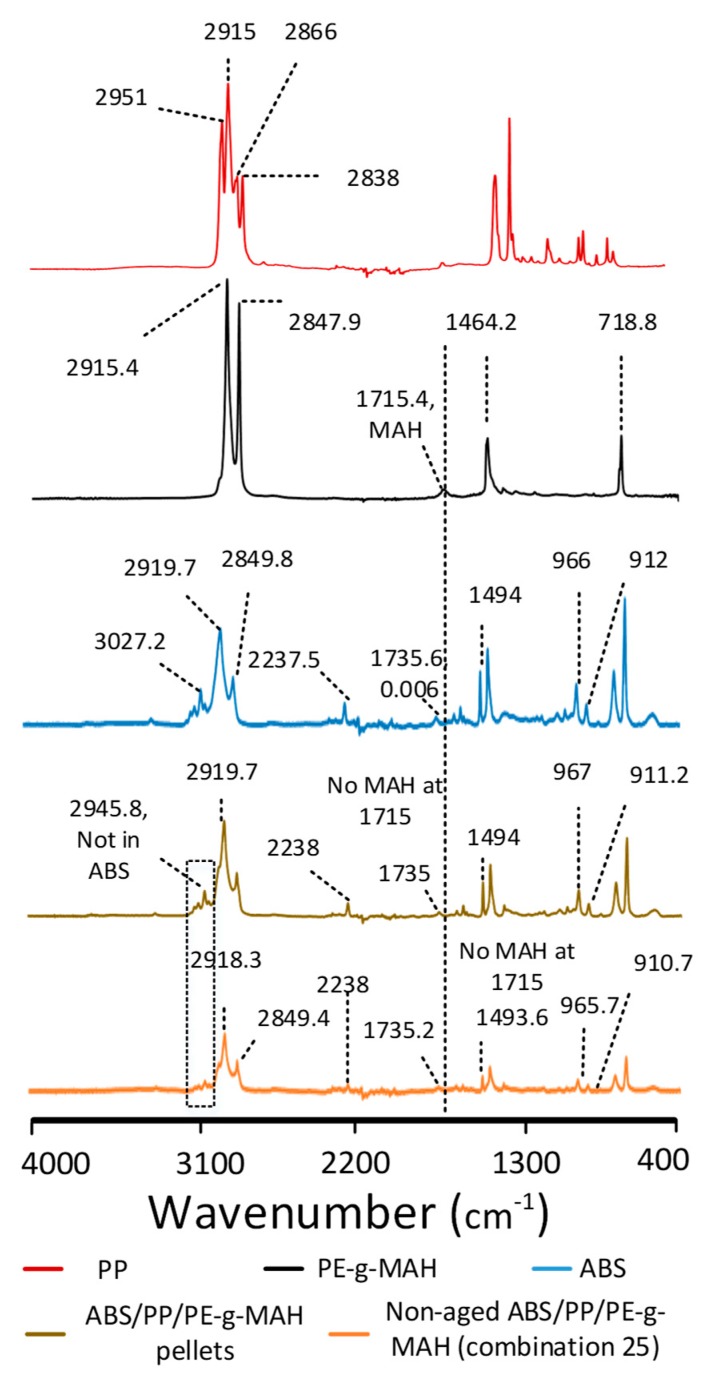
Fourier-transform infrared (FTIR) analysis of neat polymers, blend pellets, and non-aged blend.

The effects of blending, printing, and thermal aging on the intermolecular interactions as compared to neat ABS are shown in Figure 8 and Table 5. PP was noted with the asymmetric and symmetric stretching vibrations of CH_2_ and CH_3_ in the range of 2800–3000 cm^−1^, along with numerous other peaks provided in Table 5. A similar spectrum for PP was confirmed by Morent et al. [40]. PE-g-MAH was confirmed by the two C–H stretching peaks at 2914 cm^−1^ and 2847 cm^−1^, along with the MAH peak at 1715 cm^−1^ [41]. ABS was identified by acrylonitrile at 2237 cm^−1^, C=C stretching vibrations at 1637 cm^−1^, and styrene aromatic ring stretching vibrations at 1494 cm^−1^. The deformation of hydrogen attached with 1,2 butadiene at 913 cm^−1^ and 1,4 butadiene at 966 cm^−1^ [42] was also noted in ABS.

The single-screw extrusion of the polymers presented notable differences in the blend pellets as compared to the neat ABS (Figure 8). The presence of the CH_3_ group from PP [40] in the blend pellets was noted by a step merged into a wide peak at 2945.8 cm^−1^ that was absent in neat ABS. The merging of the associated group (CH_3_) of PP into the ABS blend shows the synergy [43] between the saturated hydrocarbons of three polymers. The absence of the MAH peak at 1715 cm^−1^ for the PE-g-MAH in blend pellets was also a sign of the chemical interactions [41] between the three polymers. Furthermore, it can be analyzed in Table 5 that, apart from the butadiene group at 1637 cm^−1^, all remaining groups showed high intensity as compared to the neat ABS with no shift or minimum shift. This can be interpreted on the basis of three aspects: (1) the increase in intensities for most of the groups achieved high synergy [43] to show chemical interactions, (2) the decrease of butadiene intensity showed restricted movement that could have been caused by the chemical reactions with butadiene monomers [7], and (3) the minimum shift or no shift in wavenumber presented safe blending without degrading any groups in neat ABS [7]. 

The printed blend (combination 25) presented similar peaks as found in the blend pellets but with a significant decrease in the intensities of most of the chemical groups (Figure 8). The low intensities presented a restriction of the movement of different monomers and, hence, proved the chemical interactions [7,44]. This also provided a chemical reasoning for better strength of the printed parts as compared to ABS. On the other hand, the shifts in the wavenumbers of the non-aged printed blend groups were not significant even after 3D printing. The minor (negligible) shifts in the intensities presented the unaffected quality [7,43] of printed material due to modifications in the pellet printer, such as the improved cooling system and insulation.

**Figure 9:**
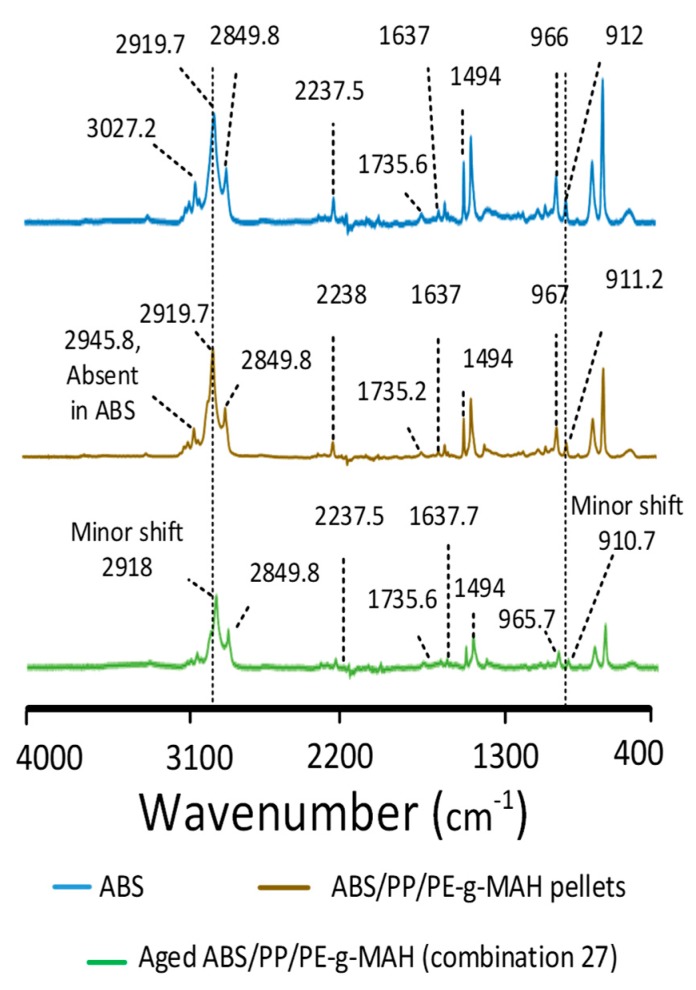
FTIR analysis for effects of thermal aging.

The analysis of thermal aging on the printed specimen (combination 27) as compared to ABS is shown in Figure 9 and Table 5. Apart from minor shifts of CH_2_ stretching [40] from 2919.7 cm^−1^ to 2918 cm^−1^ and butadiene hydrogen [7] from 912 cm^−1^ to 910.7 cm^−1^, all remaining groups were detected at similar wavenumbers. Moreover, most of the chemical groups showed a decrease in corresponding vibrational intensities. For example, the hydrogen attached with alkene carbons [7,44] at 994.6 cm^-1^ and the alkane groups [7] at 1375 cm^−1^. This described the effects of chemical interactions between the three polymers that impeded the free movement of the groups [7]. Therefore, the aging interval enhanced the chemical reactions to achieve better strength as compared to the non-aged blend.

### 4.2. Effects of Printing Variables on Crystallization 

Differential scanning calorimetry was used to obtain information about the true nature of chemical reactions in a non-compatibilized blend (ABS/PP) and pellets and the printed blend (non-aged). It was also used to investigate the reason for the decrease in strength after six days of aging in blend.

**Figure 10:**
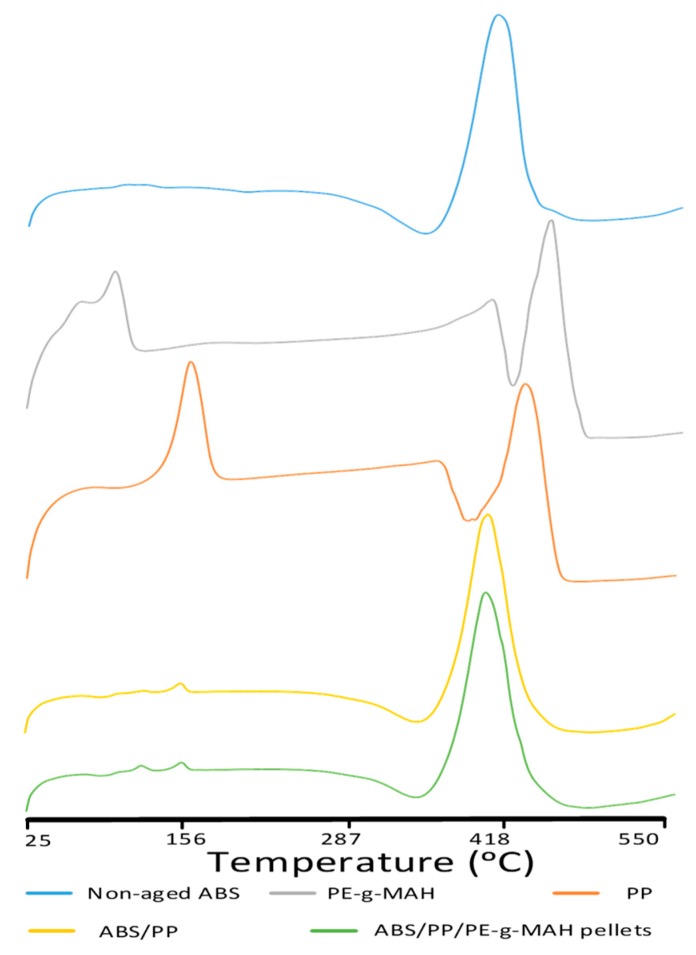
Differential scanning calorimetry (DSC) analysis of effects of melt blending.

ABS/PP showed a decrease in glass transition temperature (T_g(ABS)_) associated with ABS and in melt crystallization temperature (Tm_(PP)_) associated with PP, as compared to neat ABS (Table 6 and Figure 10). On the contrary, the increase in H_g_ (2.464 j/g) and H_d_ (488.8 j/g) in compatibilized blend pellets presented enhanced blending [46] due to PE-g-MAH. The increase in glass crystallization showed a rearrangement of intermolecular bonds at an earlier temperature [46] than neat ABS, and the high degradation energy in H_d_ presented improved resistance to thermal degradation.

**Figure 11:**
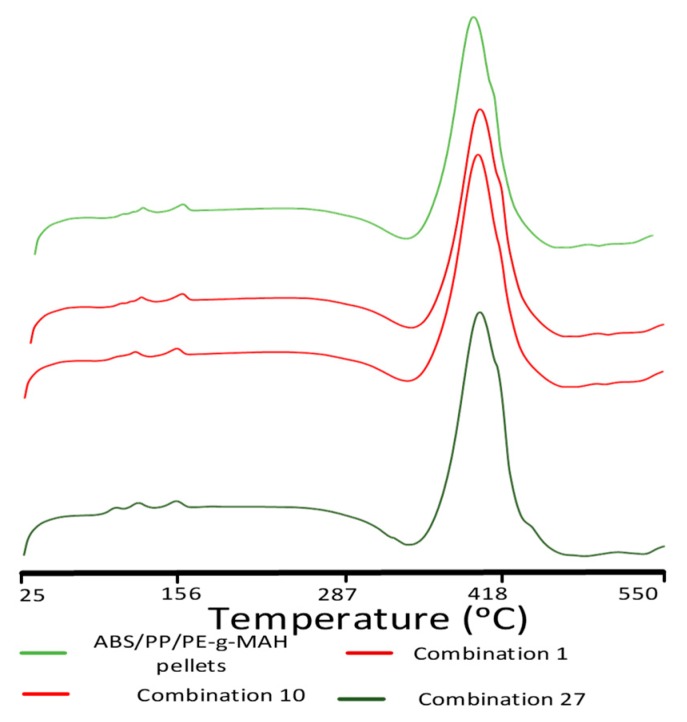
DSC analysis of effects of thermal variables.

The effect of bed temperature, printing temperature, and aging interval on thermal stability of ABS/PP/PE-g-MAH is displayed with respect to non-printed pellets in Figure 11 and Table 6. The increase in bed temperature, printing temperature, or aging interval resulted in a decrease in T_M(ABS)_ and T_M(PP)_ as compared to the non-printed pellets up until three days of aging, after which it showed a minor increase of 0.6 °C for six days of aging. The decrease may be due to the high viscosity and nucleation abilities of ABS, which help to achieve oriented crystalline polymeric chains at low temperature [47]. Moreover, the minor (negligible) increase in T_M(ABS)_ after six days of aging (combination 27) showed the high thermal stability of the material. The high thermal stability was also displayed with significantly enhanced H_d_ (>500 j/g), which was notable as compared to non-printed pellets (488.8 j/g) and neat PLA (445.8 J/g).

The absence of glass transition at 100 °C and the visible appearance of a new melt crystallization transition at 109.3 °C in the aged blend (combination 27) were significant chemical modifications due to aging. The T_M(ABS)_ at 109.3 °C was associated with ABS in the blend that occurred at 111 °C in neat ABS. However, the six days of aging resulted in a significant decrease in T_d_ and enthalpy of degradation (500 j/g) as compared to three days of aging. The appearance of a new melt crystallization peak and the decrease in corresponding enthalpies and temperature of degradation as compared to combinations aged for 0–3 days pointed toward the degradation of intermolecular bonds. Furthermore, the decrease in T_d_ and H_d_ provided a chemical interpretation for a decrease in tensile strength for samples aged for six days (combination 27) as compared to samples aged for 0–3 days. This degradation in combination 27 described the decrease in mechanical strength noted in the surface plots (Figure 5).

### 4.3. Analysis of Mass Degradation and Resistance to Thermal Aging

**Figure 12:**
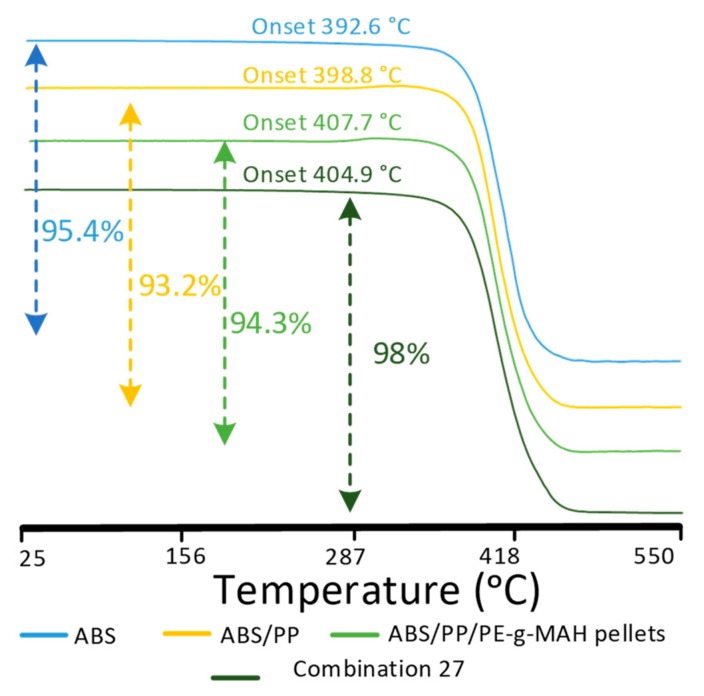
Thermogravimetric analysis (TGA) for effects of blending and thermal aging, and the percentage degradation till 550 °C.

TGA analysis is shown in Figure 12 and Table 7. The onset of neat ABS and PP are in accordance with the literature [48]. The low onset temperature of degradation of ABS was due to the hydrogen abstraction from butadiene monomers [49]. The non-compatibilized ABS/PP showed an increase in onset temperature to 398.8 °C. The increase in onset of ABS was due to the addition of PP that provides sites for partial nucleation [50]. However, the addition of PE-g-MAH as a compatibilizer in ABS/PP pellets significantly raised the onset of degradation to 407.7 °C. This significant rise in onset showed the enhanced thermal stability of the blend system [51]. The increase in onset also justified the high T_g_ and T_d_ values of DSC for compatibilized pellets.

The effects of printing parameters are also analyzed in Table 7. It can be noted that an increase in bed temperature (combination 1 to 19) resulted in a decrease in onset temperature as compared to pellets. This shows that the printing parameters affected the chemical behaviors of the blend, which was also noted in the FTIR analysis in the form of a decrease in vibrational intensities of the printed blend as compared to neat ABS. However, the differences in onset among different printing combinations were not significant, as observed in Table 7. This shows a consistent thermal behavior of the blend toward stability regardless of high temperatures and aging intervals. 

The six days of aging at the highest printing and bed temperature (combination 27) presented an increase in onset temperature (403.9 °C). Furthermore, the 50% mass degradation occurred at the highest temperature (433.3 °C) among all combinations, ending up with just 0.2% more degradation in mass (97.9%) at 590 °C. The high temperatures for onset [51,52] and the 50% degradation at the highest temperature showed the high thermal stability of the blend material as compared to neat ABS. The meager 0.2% increase in thermal degradation at 590 °C was basically the main reason for the decrease in T_d_ and enthalpy of degradation (ΔH_d_) in DSC for combination 27. This negligible 0.2% degradation was detected as a non-significant factor in ANOVA analysis. Therefore, this proves that the blend showed high thermal stability to high temperatures and aging intervals.

### 4.4. Validation of Physical Interlocking or Chemical Grafting 

SEM analysis was used to analyze the phase separation to confirm blending. It was also used to analyze the visual effects of six days of aging on fractured surfaces.

**Figure 13:**
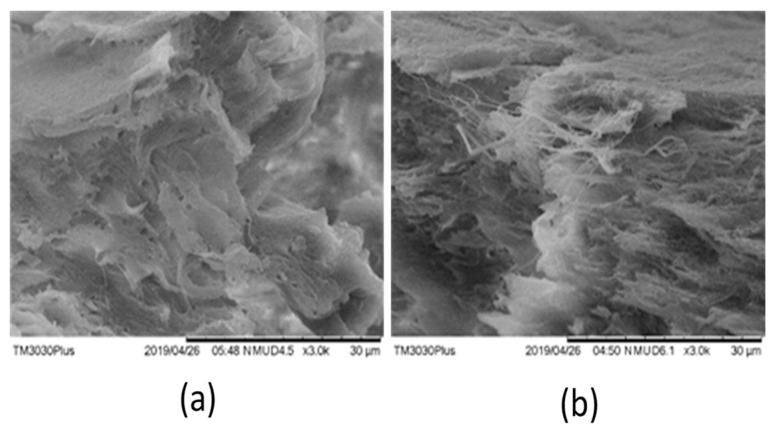
SEM images for (**a**) combination 4, and (**b**) combination 21.

Most of the SEM images showed no phase separation, as observed in Figure 13a, which shows the chemical grafting of PP on ABS through PE-g-MAH. However, there was one rare case (combination 21, sample 2) in which minor signs of phase separation were observed. Figure 13b shows PP in the form of fibers interlocked in an ABS matrix. These elongated fibers were produced out of the ABS matrix as a result of high tensile loading during breakage. The entangled fibers of PP confirmed the good tensile strain (0.0224 mm/mm) for combination 21, as shown in Table 4. The individual clusters of PP in the ABS matrix were also detected in FTIR analysis (Figure 8) as a distinct “step” at 2945.8 cm^−1^ that was merged into the wide peak of ABS saturated hydrocarbon. Therefore, the chemically grafted PP in ABS through a compatibilizer, along with localized physical interlocking, was the main reason deduced for the high thermo-mechanical properties of the novel FFF blend system.

**Figure 14:**
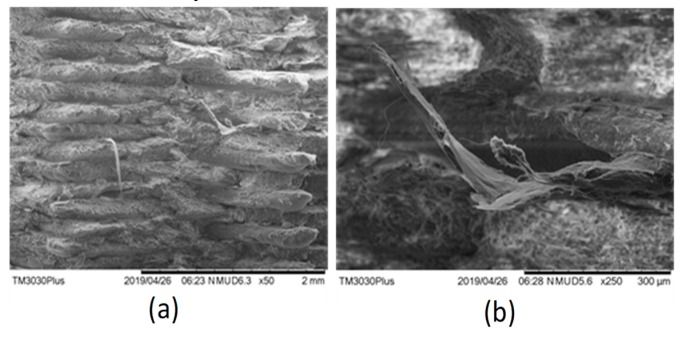
SEM images for (**a**) combination 18, and (**b**) magnified image for combination 18.

The effects of six days of aging and the highest printing temperature (205 °C) on the blend (combination 18) were observed in the form of the large pulled-out fibers after fracture (Figure 14). The enhanced ductility shown by the pulled-out fibers was also confirmed by the high strain (0.023 mm/mm) as provided in ANOVA (Table 4). This shows the ability of the novel blend to maintain at least the strength and/or the ductility in severe thermal conditions.

## 5. Conclusions

ABS is the one of the known commercial polymers for FFF. Despite its commercial benefits, it has poor thermal stability to aging at high temperatures. The degradation leads to hydrogen abstraction from the butadiene monomer of the terpolymer, which results in degraded mechanical properties. This requires ABS to have strong thermo-mechanical stability, particularly in current scenarios, where FFF is moving toward large-scale manufacturing. This research presents a novel blend of ABS with PP in the presence of a compatibilizer (PE-g-MAH) that can withstand high temperatures and a long period of thermal aging. A general full factorial experimentation with 33 factors was designed with three variables of bed temperature, printing temperature, and aging interval to analyze tensile properties. Each of the variables had three levels that were set to the minimum and maximum possible limits in the given resources. The highest bed and printing temperature-based combinations (25, 26, and 27) were selected to print samples for compression and flexure testing. The specimens of tensile, compression, and flexure were printed with a custom-designed pellet printer that was specifically modified with an improved cooling system, a thermal insulation barrier, and a refined extrusion screw with no compression zone. The modifications helped to achieve printing properties similar to an as-made blend without any chemical deterioration. The main highlights of the blend materials are provided below.

The ANOVA analysis statistically proved aging to be a non-significant factor.

The blend provides an improved tensile strength (31.6 MPa) at the highest bed and printing temperature with six days of aging (combination 27) instead of degradation, which is one of the highest mechanical properties among current FFF blends of ABS in the literature.

The blend material has higher stiffness as compared to neat ABS.

The compression and flexural properties also show a significant increase with an increase in aging interval instead of degrading.

The enhanced thermo-mechanical properties are the result of chemical grafting along with the localized physical interlocking observed in SEM.

The current work covers the broader aspects of the novel FFF material. The future work will be based upon repetitions (replicates) in the DoE of ANOVA. This will help to prove the aging interval to be non-significant with certainty.

## Figures and Tables

**Table 1:** Parameters for single-screw melt blending.

Variable	Value
Feeder to nozzle temperature	175 °C, 175 °C, 180 °C, 180 °C, 180 °C, 180 °C, 180 °C, 170 °C, 165 °C, and 150 °C
Feed rate	21 rpm
Screw speed	210 rpm
Torque	45%
Die pressure	51 bar

**Table 2:** Compositions prepared for the blend, printing bed type, and effects on printability (ABS-Acrylonitrile butadiene styrene, HDPE-High density polyethylene, PE-g-MAH- Polyethylene graft maleic anhydride).

Composition	ABS	HDPE	PE-g-MAH	Printing Bed	Printability
1	48	48	4	Perf board and adhesion tape	No
2	75	21	4	Perf board and adhesion tape	No
3	85	11	4	Perf board and adhesion tape	Yes, but excessive warpage caused separation of intercalated layers
4	92	7.5	0.5	Adhesive tape	Yes

**Table 3:** Parameters for screw extrusion three-dimensional (3D) printing.

Parameters	Values
Feed rate	5 mm/min
Printing speed	5 mm/min
Layer thickness	0.2 mm
Raster width	0.2 mm
Raster angle	45°/−45°
Infill density	100%
Multiplier	15
Number of contours	1
Nozzle diameter	0.4 mm

**Table 4:** The 3^3^ full factorial design of experiments with tensile strength (MPa) and strain (mm/mm).

Std Order (Combination)	Run Order	Bed Temperature	Nozzle Temperature	Post-Printing Aging	Tensile Strength	Tensile Strain
18	1	50	205	6	28.7	0.022
4	2	25	195	0	25.4	0.02
1	3	25	185	0	28.2	0.020
7	4	25	205	0	24.9	0.019
14	5	50	195	3	31.1	0.025
16	6	50	205	0	28.7	0.024
27	7	75	205	6	31.6	0.023
15	8	50	195	6	29.8	0.023
2	9	25	185	3	28.9	0.019
9	10	25	205	6	23.3	0.017
10	11	50	185	0	22.9	0.020
24	12	75	195	6	31.2	0.023
5	13	25	195	3	24.9	0.018
20	14	75	185	3	29.7	0.023
25	15	75	205	0	30.3	0.025
3	16	25	185	6	29.1	0.02
21	17	75	185	6	19.6	0.022
11	18	50	185	3	27.5	0.021
17	19	50	205	3	31	0.020
13	20	50	195	0	30.1	0.025
12	21	50	185	6	25	0.018
23	22	75	195	3	30.8	0.023
6	23	25	195	6	26.2	0.013
19	24	75	185	0	28.2	0.022
26	25	75	205	3	28.8	0.023
22	26	75	195	0	30.3	0.022
8	27	25	205	3	26.4	0.019

**Table 5:** Fourier-transform infrared (FTIR) analysis; WN stands for wavenumber (cm^−1^).

ABS		PP		PE-g-MAH		ABS/PP/PE-g-MAH Pellets		ABS/PP/PE-g-MAH Combination 25		ABS/PP/PE-g-MAH Combination 27		Comments
WN	Intensity	WN	Intensity	WN	Intensity	WN	Intensity	WN	Intensity	WN	Intensity	
		2950.6	0.177			2945.8	0.078	2949.6	0.024			CH_3_ stretching [40]
2919.7	0.084	2915.8	0.219	2915.4	0.404	2919.7	0.152	2918.3	0.050	2918	0.0567	CH_2_ stretching [40]
2849.8	0.041	2866.2	0.114	2847.9	0.360	2849.8	0.068	2849.4	0.025	2849.8	0.0293	
		2838.3	0.114									CH_3_ stretching [40]
2237.5	0.018					2238	0.019	2238	0.0052	2237.5	0.0059	C=N bond [42]
1636.8	0.008					1637	0.007	1637.7	0.0039	1637.7	0.005	Butadiene stretching [42]
1602.5	0.0147					1603	0.016	1602	0.0042	1602.1	0.006	
				1715.4	0.02							MAH group [41]
1735.6	0.006					1735.2	0.005	1735	0.0029	1735.6	0.0035	
1494	0.046					1494	0.053	1493.6	0.0122	1494.1	0.0155	Styrene ring
1453	0.066	1456.98	0.114	1464.2	0.113	1453	0.0816	1452	0.0205	1452.6	0.0248	
		1375	0.181			1377	0.0166	1375	0.006	1375	0.0059	Alkane CH_2_ and CH_3_ deformation, C–H asymmetric [40,45]
						994	0.0106			994.6	0.0024	Out-of-plane bending (=C–H and =CH_2_)
966	0.035					967	0.0409	965.7	0.009	965.7	0.0115	H attached to 1,4 butadiene [42]
912	0.017					911.2	0.0189	910.7	0.0043	910.7	0.0053	H attached to 1,2 butadiene [42]

**Table 6:** Differential scanning calorimetry (DSC) analysis.

Material	T_g_	T_m_	T_d_	H_g_	H_m_	H_d_
ABS	100.2	111.8 131	430.4	--	3.0 (combined peak)	445.8
PP		170.6	458		82.5	148
PE-g-MAH		108.6	475.8		27.22	164.5
ABS/PP	94.1	132.7 (ABS) 163.4 (PP)	427.5	2.251	1.09 (ABS) 4.6 (PP)	489.5
ABS/PP/PE-g-MAH pellets	93.5	128.7 (ABS) 163.6 (PP)	424.8	2.464	3.61 (ABS) 2.72 (PP)	488.8
ABS/PP/MAH combination 1	91.6	127.7 (ABS) 163.7 (PP)	425.9	2.53	1.69 (ABS) 4.075 (PP)	525.6
ABS/PP/MAH combination 10	93.8	127.4 (ABS) 163.5 (PP)	426.1	2.502	2.93 (ABS) 4.39 (PP)	529.4
ABS/PP/MAH combination 27	--	109.3 (ABS) 129.3 (ABS) 163.9 (PP)	427.1		1.37 (ABS) 2.86 (ABS) 4.03 (PP)	500

**Table 7:** Thermogravimetric analysis (TGA).

Polymers	Onset Temperature	Degradation	
		Temperature at 50% Mass Degradation (°C)	Mass Degradation at 590 °C (%)
**PP**	409.1	457.8	100
ABS	392.6	436.1	95.4
ABS/PP	398.8	433.3	93.2
ABS/HDPE/PE-g-MAH pellets	407.7	433.7	94.3
ABS/HDPE/PE-g-MAH Combination 1	403.8	432	98.18
ABS/HDPE/PE-g-MAH Combination 10	403.5	431.6	97.7
ABS/HDPE/PE-g-MAH Combination 19	402.7	429.3	97.7
ABS/HDPE/PE-g-MAH Combination 27	403.9	433.3	97.9
